# “Could You Work in My Team?”: Exploring How Professional Clinical Role Expectations Influence Decision-Making of Assessors During Exit-Level Medical School OSCEs

**DOI:** 10.3389/fmed.2022.844899

**Published:** 2022-05-06

**Authors:** Bunmi S. Malau-Aduli, Richard B. Hays, Karen D'Souza, Karina Jones, Shannon Saad, Antonio Celenza, Richard Turner, Jane Smith, Helena Ward, Michelle Schlipalius, Rinki Murphy, Nidhi Garg

**Affiliations:** ^1^College of Medicine and Dentistry, James Cook University, Townsville, QLD, Australia; ^2^School of Medicine, Deakin University, Geelong, VIC, Australia; ^3^School of Medicine, Notre Dame University, Chippendale, NSW, Australia; ^4^School of Medicine, University of Western Australia, Perth, WA, Australia; ^5^School of Medicine, University of Tasmania, Hobart, TAS, Australia; ^6^Medical Program, Bond University, Gold Coast, QLD, Australia; ^7^Adelaide Medical School, University of Adelaide, Adelaide, SA, Australia; ^8^School of Medicine and Health Sciences, Monash University, Melbourne, VIC, Australia; ^9^Medical Program, University of Auckland, Auckland, New Zealand; ^10^School of Medicine, University of Sydney, Sydney, NSW, Australia

**Keywords:** medical education, clinical assessment, cultural historical activity theory, objective structured clinical examinations, prototypical intern

## Abstract

Decision-making in clinical assessment, such as exit-level medical school Objective Structured Clinical Examinations (OSCEs), is complex. This study utilized an empirical phenomenological qualitative approach with thematic analysis to explore OSCE assessors' perceptions of the concept of a “prototypical intern” expressed during focus group discussions. Topics discussed included the concept of a prototypical intern, qualities to be assessed, and approaches to clinical assessment decision-making. The thematic analysis was then applied to a theoretical framework (Cultural Historical Activity Theory—CHAT) that explored the complexity of making assessment decisions amidst potentially contradicting pressures from academic and clinical perspectives. Ten Australasian medical schools were involved with 15 experienced and five less experienced assessors participating. Thematic analysis of the data revealed four major themes in relation to how the prototypical intern concept influences clinical assessors' judgements: (a) Suitability of marking rubric based on assessor characteristics and expectations; (b) Competence as final year student vs. performance as a prototypical intern; (c) Safety, trustworthiness and reliability as constructs requiring assessment and (d) Contradictions in decision making process due to assessor differences. These themes mapped well within the interaction between two proposed activity systems in the CHAT model: academic and clinical. More clinically engaged and more experienced assessors tend to fall back on a heuristic, mental construct of a “prototypical intern,” to calibrate judgements, particularly, in difficult situations. Further research is needed to explore whether consensus on desirable intern qualities and their inclusion into OSCE marksheets decreases the cognitive load and increases the validity of assessor decision making.

## Introduction

The assessment of clinical, communication, and practical skills is an important component of health professions education for both feedback provision and informing progress decisions. Learners are placed and observed in a variety of settings—teaching facilities, simulation centers, and “real world” practice—to ensure that learning outcomes are achieved. Expectations of learners evolve through programs, with the focus changing from individual components (e.g., taking a history, measuring blood pressure, examining a body system) to more integrated comprehensive tasks that require focus on presentations, diagnostic reasoning, and management plans. Marking sheets may include checklists, rating scales for individual items or competencies, global scores and free-text comments. Assessors are a diverse group, including people in various combinations of academic and clinical roles, in different clinical specialties, and with different levels of experience as clinicians and assessors. Their assessments often rely on relatively brief observations of performance to inform judgements, drawing on both stated learning outcomes and their own experiences and expectations of good clinical performance (1).

Significant variability in clinical assessor judgements has been reported (2–5). However, variations often persist despite assessor training and standardized station design (6–8), raising concerns about cognitive bias in assessor judgments. Cognitive biases, also known as “heuristics,” are cognitive short cuts used to aid our decision-making (9). Studies have shown that clinical decision-makers are at risk of error due to bias but often lack insight into their own biases (10). There are various causes of bias, and these include learned or innate biases, social and cultural biases, and environmental stimuli (11). This highlights a need for greater understanding of the cognitive processes of clinical assessors to inform strategies that enhance fair and robust judgements. Our previous research showed that judging candidate performance is complex, cognitively challenging and mentally demanding, particularly when borderline performance is observed in an “exit” Objective Structured Clinical Examination (OSCE) (12). In this “grey” zone of candidate performance, assessors used academic institutional marking criteria as a “safety blanket” to guide judgement, but also used additional criteria that were not necessarily explicit in the marking sheet, based on professional expectations (candidate demeanor and patient safety). The emergence of the concept of a “prototypical” intern (in Australia and New Zealand, this is a first postgraduate year medical graduate working under supervision in teaching hospitals) suggested that calibration was guided by a rapid, internal cognitive process based on a mental construct of assessors' expectations of a new medical graduate working in their clinical team.

In educational psychology this construct is known as a heuristic cognitive process, conscious or unconscious, whereby “rule of thumb” judgement decisions are made, possibly neglecting some presented information (13–15). This mental “shortcut” is faster and reduces cognitive complexity when working memory is overloaded by time pressure or increased complexity and is also more likely in experienced assessors who recognize patterns more quickly (14, 16, 17). Applying this concept to our previous study, assessors appeared to use a representativeness heuristic to consider “how much does the observed clinical performance of a senior medical student compare with what I expect of a ‘prototypical’ intern” (12)? Such representational heuristics may be influenced by assessors' roles and experiences, contrast effects, use of inference, working memory effects, different interpretations of behaviors, predisposition to consider a particular perspective (e.g., of the learner or patient), different pre-existing frames of reference, exposure to different learner cohorts, and the examiners' own clinical skills and perceptions of task difficulty (18–21).

So far, exploratory studies on assessor cognition have focused mainly on workplace-based assessments (WBAs), usually involving learners performing authentic tasks in clinical settings (5, 22–25). These studies consistently find that assessor judgements are complex (5, 23, 25–27). Examiner decision-making in OSCEs has been less researched, yet OSCEs remain a popular clinical assessment format despite the trend toward WBA and the challenges imposed by the recent COVID-19 pandemic. Whereas, WBA requires assessors to interpret the clinical case and set specific expectations within a marking framework, OSCE assessor judgements are guided by prescribed expectations and scoring criteria provided via the mark sheet by educators for more time-limited, standardized and pre-scripted encounters. This may require assessors to adapt their expectations of learner performance according to situational constraints. This paper reports a further exploration of the cognitive processes of exit level OSCE assessors with a primary aim of exploring how the prototypical intern concept influences clinical assessors' judgements in senior medical student OSCE. The secondary aim was to explore the complexity of making OSCE assessment decisions amidst potentially contradicting pressures from academic and clinical perspectives. Two specific research questions have been developed to address these aims—(1) How does the prototypical intern concept influence clinical assessors' judgements in senior medical student OSCE? (2) how do OSCE assessors balance academic (focussing on achieving graduate outcomes) and professional (being suitable for work in a clinical team) expectations when assessing senior medical students?

## Methods

### Study Context and Design

This research was informed by the interpretivist paradigm, which is a relativist ontology with a subjectivist epistemology (28). Interpretive paradigm focuses primarily on recognizing and narrating the meaning of human experiences and actions (29). In the interpretive paradigm, knowledge is relative to particular circumstances (historical, temporal, cultural, subjective) and exists as multiple interpretations of subjective experiences of reality (28). Empirical phenomenological qualitative approach was the methodology used to explore the concept of a “prototypical intern” from the assessors' perspectives (30). Phenomenology aims to explain the nature, essence and veracity of phenomena with the aim of understanding the complexity of the participant's lived experiences (31). Empirical phenomenology produces explanations that are grounded in the subjective experiences of the participants with an understanding of why and how things happen (30). This is expressed as a theory—a set of interrelated concepts that must be grounded in the meaningful experiences of the participants studied (30, 32).

We focused on exit-level OSCEs in Australian and New Zealand medical schools within the Australasian Collaboration for Clinical Assessment in Medicine (ACCLAiM) network. Their medical programs are mapped to a national medical graduate competencies framework and have similar integrated, outcomes-based curricula and OSCE processes, including some shared stations (33–35). This study was approved by the James Cook University Human Research Ethics Committee (H6833) and accepted by all participating universities.

### Participant Recruitment

All thirteen ACCLAiM member schools were invited by email in November 2020 to purposefully recruit both experienced and less experienced assessors from their OSCE examiner pools. For consistency, an experienced assessor was defined as having five or more years of post-specialty training clinical practice, experience in assessing senior medical students and/or junior doctors, and a record of consistent and reliable scoring. Ten schools agreed to participate, providing a list of 39 assessors (25 experienced, 14 less experienced) who were invited by email to participate, supplied with an information sheet and provided written consent.

### Focus Group Sessions

Focus group discussions (FGDs) were conducted to enhance exchange and clarification of participants' viewpoints by exploring how and why they think in a particular way (36). Semi-structured questions (see Supplementary Material 1) were developed by the research team based on the literature and their experience, focusing on responses and ideas surrounding the “prototypical intern” concept (12). FGDs had durations of 45–60 min and were conducted between December 2020 and April 2021, hosted on an online video-conferencing platform and facilitated by three of the authors (RBH, BMA and KDS). Sessions commenced with verbal confirmation of consent. Questions were used to open discussions or probe emerging issues more deeply. Discussions were recorded and transcribed verbatim by a professional transcription service. Participants were de-identified and differentiated by sex, level of examining experience and a participant ID. Data collection and analysis occurred concurrently and ceased after five FGDs as responses were no longer revealing new information.

### Data Analysis

There were two stages of analysis. The first involved the use of inductive thematic analysis with emerging themes identified, based on the tenets of Braun and Clarke (37). This was aimed at understanding how the prototypical intern concept influences clinical assessors' judgements in senior medical student OSCE. The second stage involved the application of the findings from the thematic analysis to a theoretical framework that could explore the complexity of making OSCE assessment decisions amidst potentially contradicting pressures from academic and clinical perspectives. This analytical approach aided the positioning and contextualization of an applicable theory into the research (38).

We chose the third-generation cultural historical activity theory (CHAT) developed by Engeström (39, 40) because it provides a robust framework for analyzing professional work practices (41). CHAT has been applied widely in education research (42–46) and in medical education to investigate students and health professionals' knowledge of patient safety (47), patient care (48–50) and the consistency of OSCE examiner judgements and implications for examiner training (51). The framework has also been utilized to explore the authenticity of OSCEs, their impact on learning and the judgements of WBA assessors (48, 52).

The value of CHAT is that it develops “conceptual tools for understanding dialogue, multiple perspectives and voices, and networks of interacting activity systems” (53), centered on three core ideas: (1) humans act collectively, learn by doing, and communicate in and via their actions; (2) humans make, employ, and adapt tools of all kinds to learn and communicate; and (3) community is central to the process of making and interpreting meaning—and thus to all forms of learning, communicating, and acting within the context of a community (39, 54–56). This facilitates a systematic, multi-dimensional approach for exploring a comprehensive set of dynamic factors (41) that in this study relate to assessor judgements. The primary unit of analysis is an activity system (39), a network of sociocultural elements, with complex mediational structures, that shape the collective actions of individuals who are motivated to achieve a goal (57–59). The common elements within an activity system are subject, object, instrument, outcome, rules, division of labor and community (39, 54). The framework explores interactions between each of these elements both within and between two Activity Systems, (AS) (48, 52) which were assigned as academic (AS1) and clinical (AS2).

Transcribed data were analyzed according to framework elements using NVivo version 12 software (QSR International, Melbourne, Australia). This approach utilizes both inductive and deductive analytical techniques and entails six stages: (1) Reading and re-reading the textual data to familiarize oneself with the content, (2) Identifying, devising, or refining a thematic framework to facilitate data analysis, (3) Indexing the data to corresponding themes, (4) Charting the identified themes (5) Mapping, and (6) Interpreting the themes generated (60). The coding process described by Meijer et al. (50) was utilized in which codes belonging to different parts of the CHAT-model and contradictions within and between the AS were created, reviewed and, if necessary, revised throughout the analysis. The coding process was completed by two authors (BM-A and RH), confirmed by two other authors (SS and KD'S) and discrepancies were resolved in a consensus meeting.

## Results

### Participants' Characteristics

A total of 20 assessors participated in the five FGDs, each with between 3 and 6 participants. The number of participants per group was kept low to foster rich FG discussions. There were 7 females and 13 males with, respectively, 8.4 and 14.8 mean years of clinical experience (range 1–45 years). Fifteen were experienced assessors (5 females and 10 males) and 5 less experienced (2 females and 3 males), almost all in dual roles as academics engaged in medical education as well as clinical practice. Less experienced assessors were all clinicians, although one also held an associate lecturer role. All participants were coded by their level of experience (Experienced or Less experienced; Exp or Less), sex (Male or Female; M or F) and an individual participant number. Details of the participants' characteristics are shown in Supplementary Material 2.

### Thematic Analysis Findings

Thematic analysis of the FGD transcripts revealed four major themes in relation to how the prototypical intern concept influences clinical assessors' judgements in senior medical student OSCEs. These themes are (a) Suitability of marking rubric based on assessor characteristics and expectations; (b) Competence as final year student versus performance as a prototypical intern; (c) Safety, trustworthiness and reliability as constructs that require assessment; and (d) Contradictions in decision making process due to assessor differences.

#### Suitability of Marking Rubric Based on Assessor Characteristics and Expectations

Participants demonstrated a good understanding of their roles as OSCE assessors within AS1 and the requirement of compliant use of the marking criteria proforma to assess students' clinical performance.

“For the OSCE, from my experience, there's a proforma, there's specific questions and there's marking attached to it, and then there's always a clear description. So essentially, when I'm assessing a student using that proforma, I will follow what's written” Exp-F-P09

Participants reported knowing that they had to complete both the checklist and global rating scales of the marking rubric and claimed to understand the rationale for adhering to the criteria, despite sometimes experiencing a personal cognitive dissonance with the rubric.

“I feel like my job as an assessor in OSCEs is to follow the assessment sheet and follow the criteria fairly closely. And I have been in situations when I've felt that the assessment criteria didn't necessarily reflect what I would expect an intern to be capable of. And so, have provided feedback after the assessment that I felt that, you know, perhaps the assessment criteria, were more pitched at a fourth-year level rather than a fifth-year level or something like that. But I felt that because their criteria for consistency across examiners that it's really important to stick to them.” Exp-F-P07

However, when engaging with the marking criteria to assess the students, participants also believed that the listed criteria did not necessarily reflect all aspects of the expected performance. At this point, they felt that there were relevant elements of subjectivity from their clinical experience, and they had their own personal views about expected standards that related to their clinical work environment.

“But even just reflecting on our OSCE about a month ago, yeah, examiners still have different views, personal views about what they think the minimum standards should be, despite how the committees…adjudicate as to what is the minimum standard. So, there's always going to be some difference there.” Exp-M-P05

#### Competence as Final Year Student vs. Performance as a Prototypical Intern

The concept of the “prototypical intern” was applied as the assessors began to critically appraise and compare the final-year medical students' performance to that of their junior doctors/interns in the clinical work environment. For most assessors, the comparison between the final year medical student and the prototypical intern happened intuitively, where the student was mentally placed into the AS2 environment for evaluation.

“When we're assessing in that final year in the clinical assessments, to my mind, we are explicitly telling students and other examiners, that the level we are setting the assessment at is: will you in the next few months be able to work as a PGY1 doctor, and be able to give me a reasonable differential diagnosis and some initial management steps?” Exp-M-P13

“When I think of how to examine these students, I do also think as to what I expect…a standard intern to be. It's a bit easier because I was an intern two years ago. So, it's quite fresh in my mind as to the standard that I was at, and what my peers were at. And then last year, for six months, I was a tutor for sixth year medical students. So, I had a good grasp as to what level they were at, as they were just reaching the end of their internships. And so, looking at that cohort, and teaching them each week, I was able to know what I thought the average graduate should be at.” Less-F-P15

The concept of the prototypical intern was used to make judgements at all levels of performance—excellent, borderline or failing student. Interestingly, the ideal of a prototypical intern has been around for a long time.

“My views are pretty similar to Exp-M-P14. And they probably began about 30 years ago, when I was an undergraduate at XXX university. And we were told…as we neared the final examinations, the way we would be assessed is as an intern, and we would not pass if we harmed or killed the patient. Otherwise, we're pretty well, right. And that's something that has probably stuck with me all the way along. I tend to use it, if I'm assessing someone, really for a fail. And I say, “Okay, have they caused harm to that patient, as an intern, or has it been worse?”. And if that is the case, which is rare, but if it is, then I'll give a clear fail. If I think it's something that, you know, can be addressed, and requires some, you know, remedial education or something, then I know that a borderline is going to get them a supplementary exam and they can study harder. So, for me, it's similar. But it's the distinction between a clear failure as regards to pass/fail” Exp-M-P16

“So when I mark the student, I would think of an intern that is safe, and minimally competent, at the level, and above expectation, is obviously above the level of intern, a minimally competent intern.” Exp-M-P06

However, one assessor did not support the idea of using the prototypical intern as the yardstick, finding it unfair for final year students who had yet to experience the intern year.

“The only fair way we can assess them is where they are in the course. I don't think there's any way we can be building into our system, some second guessing about…what they're going to be like in a year's time. That's just not fair. I think the only way it's fair to do that is to say, this is a fifth-year medical student, and you judge them at the level of your expectations of a fifth-year medical student. All sorts of things could happen in the next year.” Exp-M-P2

Other assessors agreed that it was unfair to judge final year medical students at the level of an intern but attempted to provide a rationale for doing the comparison and described seeking evidence of foundational learning on which internship performance could be constructed.

“And so I think when it comes specifically to final year fifth year XXX University OSCEs, I do have that picture of when they're that first day intern, how will …this intern do? When it comes to final year OSCEs, I feel like I interact with a fair number of interns in ED, and I kind of have this idea of what an intern should know when they first come onto the floor.” Less-F-P18

“Yeah. But if you're looking at things that would concern you about a student progressing, okay, I agree with the notion that we're not looking for perfection, but we're looking for evidence, if you like, of something that's already there, rather than something that might need to be added on in turn, and I'm thinking more of a skill rather than content” Exp-M-P01

#### Safety, Trustworthiness, and Reliability as Constructs That Require Assessment

Assessors often looked for the professional behavior characteristics of a good intern in candidates' performances. While most of these qualities are not readily assessable in an OSCE, and thus tend not to feature in marking rubric, they still influenced examiner judgements. The criteria that assessors considered during their judgements included important professional behaviors such as good communication, safe practice, trustworthiness, reliability, and insight into one's own limitations and scope of practice.

“We're all wanting that registrar, or that student, or an intern whose knowledge isn't as good. But we'll trust them, we'll be more confident with them. If the knowledge isn't good, then that's easily fixed. If they're not trustworthy…then that's really an issue. But how do you test that in an OSCE?” Exp-F-P03

“I tend to be looking at qualities in a student and that hypothetical intern that I have in mind is the one that I would trust, to work for me. And I'd be able to trust the information they were giving me; I'd be able to trust that they got the information that they should have got. And I would be sort of, I'd be able to rely on that. And I'd expect to then go and see the patient with them or separately, and to be able to see something that was consistent with that.” Exp-M-P01

It was recognized that unconscious positive bias toward candidates of perceived similarity to self may also influence assessor judgements, as a cognitive overlay to the prototypical intern construct.

“I imagine we, even if it's just subconsciously, make judgements on how they dress, how they walk, how they talk, how much they're like us because we will probably favor them positively if we think they're like us. How well they interact with the patients, how polite we feel they are, what it is that makes them safe or what makes them unsafe, how prepared we feel they are all probably goes into whether or not we think that they're a responsible and trustworthy candidate.” Less-F-P18

Assessors also acknowledged the role of other health professionals and health service consumers in the AS2 system and integrated these perspectives into their judgements of candidate performances. The assessors expected students to demonstrate an understanding of the professional and interprofessional relationships that exist within the clinical environment. Less experienced assessors, in particular, valued the input of the simulated patients (SP) in making judgement about students' performance.

“I always also try to get a gauge of what the SP or the patient felt overall as well to see their thoughts because you know they've been doing this for years and they've seen many different students compared to us, that's also a good gauge I think.” Less-M-P19

“Where a lot of their problems come from is, as Less-M-P17 alluded to, [when they think]“Well, I'm the doctor.” They won't listen to the nurse; they won't escalate things.” Exp-M-P16

#### Contradictions in Decision Making Process Due to Assessor Differences

Assessors identified some areas of conflicting views. This mainly related to the conflicting expectations between activity systems. The assessors felt that faculty is focused on performance on the day, while assessors are more concerned with the general character of the final year medical students and their suitability to fit into the medical team in the clinical environment.

“One of the things that our [postgraduate specialty] college exam stressed was to avoid the term borderline, because it allowed the examiner to sit on the fence. And so, we tried to make the examiner be a little bit more specific, just below or just above, but don't use the term borderline. And that's something that I've held, personally, in assessing a student's overall competency. Try and work out what it is that makes them safe or what makes them unsafe but avoid borderline.” Exp-M-P05

“We use below expectation [and] at expectation. And that's really interesting. I hadn't reflected on this before. But I think that does bring in what's the expectation? So, if you're using that sort of language, it probably does encourage [if] the expectation is intern level or…graduate, ready to graduate into final year. So, I hadn't thought about that before. But now I think about it when I see those words in a marking rubric for the global score, it does point my attention to what's my expectation? So, it probably does encourage the use of the prototypical intern.” Exp-F-P07

Participants also flagged possible differences between male and female assessors as well as junior and experienced assessors; assessors marking stations inside or outside of their particular discipline and among postgraduate specialty college assessors.

“And one of the things that I've had to do in my [postgraduate specialty] College job, as chair of examiners, is to actually sit with the cohort of examiners as a co-examiner to see what the discrepancy is. And you are absolutely right that the younger examiners, and often the women, the younger women examiners are tougher than the old men. And it really is something I think examinations have to take into account that the old retirees often [have] soft touches. And they will, because of their experience of having seen mistakes made and rectified, they will be a bit more lenient. So, I think we do have to be careful about examiner variability.” Exp-M-P05

“But one of the interesting things we're finding is that it's more junior examiners, who might only be three or four years out themselves that seem to have much higher standards of the students. And those of us who've been around for a bit longer seem to be a little bit more willing to tolerate maybe poor performances.” Exp-F-P04

“We've got all sorts of examiners and obviously some [postgraduate specialty] colleges and disciplines rely more on OSCE than others. And I do notice that the colleges who do use OSCEs if we have some of their examiners who are familiar with the college exams come to examine, they tend to be a bit more hawkish. So, they do, I think, bring their expectations over. I think it's almost unavoidable.” Exp-M-P10

“At medical school level, it's sometimes difficult to get examiners who have enough knowledge of the subject to do a comfortable OSCE. I'm comfortable with general medicine, I'm comfortable with pain medicine. I am a neurologist. And I've noticed that my co-examiners who are not neurologists let some things through that I would object to.” Exp-M-P05

“It's difficult to get the examiner line-up the level that we are expecting. So obviously, we have the specialists in that particular discipline doing the examination in that OSCE station, they will expect more as Exp-M-P05 mentioned… because they are the specialists in that discipline. And they will always argue saying, but the student won't be exposed to the same scenario in year four, so, we need to set the bar high enough to be an exit-level." We will then remind them, the student will still improve their communication skills, their history taking skills, examination skills.” Exp-M-P06

To improve the examiner decision making process, the participants suggested the use of calibration sessions and well-defined performance descriptors.

“Great descriptors are really important. For example, I'm working in intensive care [and] don't have interns. They have to be PGY [postgraduate year] two, three, and have to be good to get to ICU. So, without good descriptors, I probably wouldn't know what the expected level is going to be.” Exp-F-P09

“We have a breakout for each station and all the examiners on one station have anywhere from 30 to 40 minutes to discuss that station to go through it in detail. And usually, we have the person leading that as one of the academics who has helped at least quality assure, if not write, the OSCE so that it can be standardized, and you can thrash out some of those questions about what we're actually looking for. And I think that's a useful approach.” Exp-F-P04

“Perhaps having kind of explanatory dot points as to what an excellent candidate would be. And then you know what [a] very poor or definite fail is…Then the examiner can …they had a few excellent qualities, but they did miss some, and therefore I'll put them…not quite there. Rather than explaining each individual one, just giving them a bit of a sense as to… what they could do to move them further in one direction than the other.” Less-F-P15

### Application of CHAT: Interactions Within and Between the Activity Systems

**Figure 1:**
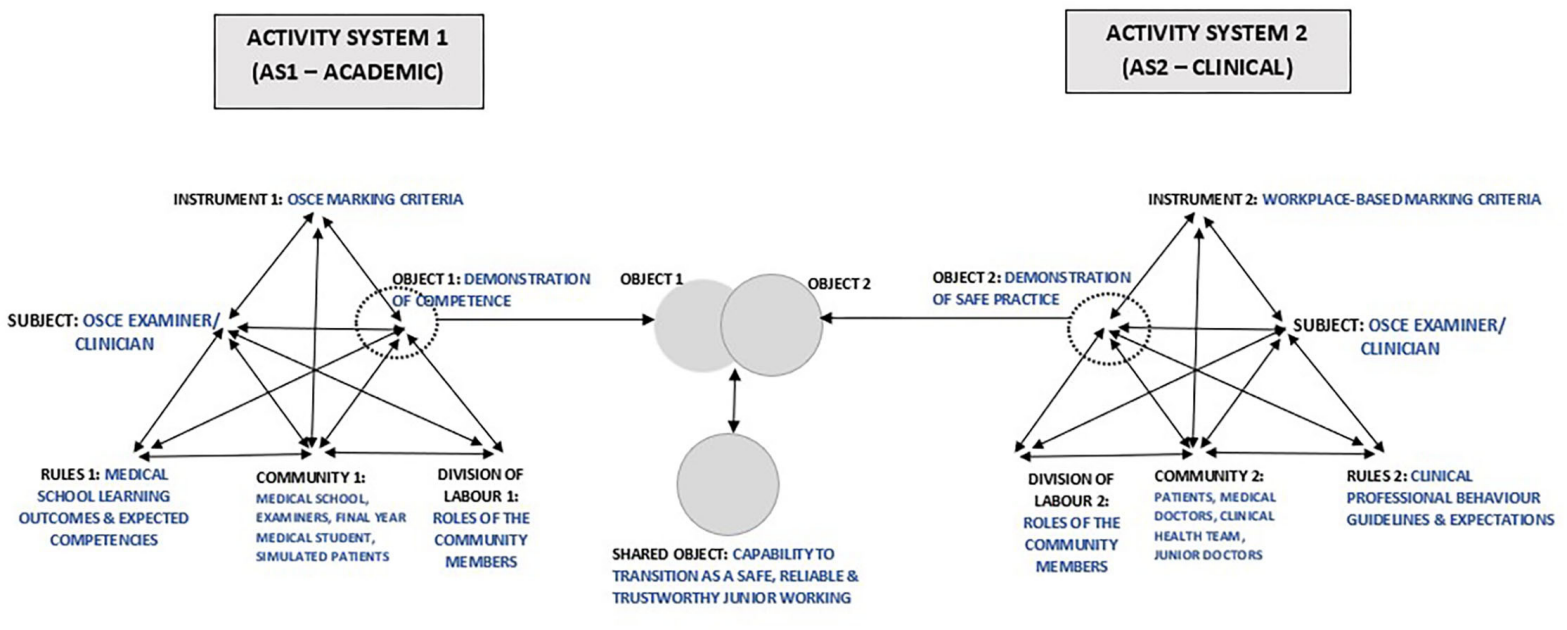
CHAT in the final year clinical OSCE.

The identified themes mapped well within the interaction between two proposed activity systems in the CHAT model: academic and clinical. In relation to how the CHAT can further explain and structure these perceptions, we present OSCE assessor judgements as an interaction within and between two activity systems (AS)—AS1 “academic” and AS2 “clinical,” which function independently but collaborate to produce an outcome. The interactions commenced in AS1 with the Subject (assessor) engaging with the marking rubric (Mediation Tool 1), provided by the academic faculty (Community 1), based on internal interactions with faculty requirements (Rules 1) and the organization of the community (Division of Labor 1), with the aim of facilitating judgement on demonstration of competence (Object 1) by final year medical students. At this point, should the assessor feel there is a misalignment of the marking criteria (Instrument 1) with their expected performance standards from AS2 elements (Object 2—demonstration of safe clinical practice, Rules 2; Community 2; Division of Labor 2; and Instrument 2), the subject (assessor) proceeds to mentally adjust Instrument 1 (OSCE marking criteria) to create a shared object—capability of the medical student to transition as a safe, reliable and trustworthy junior member of the clinical team, based on the concept of the “Prototypical Intern” that is better aligned with their expectations. Figure 1 summarizes the application of CHAT to our thematic analysis.

The academic AS1—the medical program—focuses on curriculum development and delivery, specific learning outcomes, assessment tasks, mark sheets and progress decisions, whereas the clinical AS2 focuses on global application of knowledge and skills in authentic clinical practice. The activity system of authentic clinical practice is different in almost every respect to the academic system, but assessor decision-making requires an interaction between two AS because learners are transitioning from one to the other and the assessors work in both systems. The subject in the clinical practice AS is a practitioner whose object is to care for patients. However, when this practitioner is called into the academic AS to serve as an assessor, the object in this AS is to observe and make progression judgement on the level of competence demonstrated by learners. These manifestly divergent goals/ objects in the two activity systems create tensions and contradictions for the subject. While the OSCE is focused on written rules of standardization as indicated in the marking rubric, the assessors prefer to focus on unwritten but fundamental rules and values that are core to authentic, safe and trustworthy clinical patient care.

## Discussion

This study uses the CHAT as the theoretical framework to contribute new knowledge to understanding the decision-making processes of assessors during clinical assessment of candidates at a major professional transition point—graduating from a primary medical degree course to entering the workforce. Clinical assessors make judgements that combine, to a varying extent, two interacting sets of roles and experiences that have different origins. The academic construct is the achievement of agreed, expected, graduate learning outcomes; in contrast, the clinical workplace construct is the ability to successfully work within professional settings. The former is reliant on knowledge, skills and behaviors represented in marking sheets in several scheduled, controlled assessment events, one of which is the OSCE. Academic progress decisions are informed by combining assessment data from many assessment events, converting a series of “snapshots” to a “low frame-rate” moving image. The clinical workplace construct is reliant on respect, organization, reliability, teamwork, and trustworthiness exhibited over time. Clinical assessors form impressions of what constitutes a “safe” junior doctor, a “prototypical intern,” through personal clinical experience of working with graduates. These impressions may be shared by close colleagues but different to those in other fields of health care, producing a form of cognitive bias that is likely based on a combination of “signal” and “noise.” This may provide an implicit set of “rules” that reflects “noise” and necessitates adaptation of the academic marksheet. When candidate performance is “borderline,” time is pressured or the assessor is more experienced, this characteristic heuristic, “how would this person fit into my clinical team?” is likely to be relied upon as a benchmark, either consciously or unconsciously, merging rules and influencing resulting judgements. The application of CHAT explained the intricacies and contradictions that may occur within OSCE assessor decision-making.

This study explains how assessors in certain circumstances fall back on the clinical workplace experience to influence judgements about candidates. The extent of the influence may depend on the balance of academic and clinical experiences of individual assessors. Predominantly, clinical assessors may respect the academic rules, but over-ride them or merge them with clinically informed rules. This may explain why some assessors add components to checklists, add data points to rating scales and provide negative feedback to faculty on marksheets content. Another position may be that the more academic and less clinically relevant the marksheet, the more assessors may follow clinical logic in making decisions on clinical performance—overriding the marksheet criteria. The dominance of one or other activity system may also explain why examiner training appears not to be effective and why “doves” and “hawks” are difficult to change. “Debiasing” training is becoming popular to improve clinical reasoning, but success may be elusive (61). It may also explain the popularity among clinicians of the concept of Entrustable Professional Activities (EPAs), which adopts a more holistic view of performance (62). Of particular interest is that assessors strongly held the notion that clinical performance was more than just the sum of its parts, and that professional behaviors and identity (e.g., trustworthiness, safety, and reliability) were the most highly valued attributes in candidates—over and above just the taught content. Further, assessors believed that these attributes could be judged in an OSCE station. While our participants framed their “prototypical intern” characteristics around some relatively objective professional traits such as safety, other traits e.g., trustworthiness introduce the possibility of an underlying source of cognitive bias. Halo effects derived from superficial perceptions of the similarity of candidates to assessors may be able to falsely inflate feelings of trustworthiness and thus increase the inter-rater variability when using the construct. Additionally, the assessors were seeking and interpreting performance evidence based on their existing beliefs and expectations within the heuristic of the “prototypical intern” during the OSCE, implying confirmation bias (63). Future research exploring the finer details of the individual components of the prototypical intern construct e.g., what constitutes “trustworthiness,” would provide greater insights into these potential sources of bias.

The relevance of this theory to other forms of clinical assessment, or clinical assessment at course levels other than the graduating cohort, cannot be determined from this study. The analogy may be stronger for exit assessments for other health professionals, where clinicians are assessors in an OSCE-like event. The model may be less relevant to WBA, where the clinical workplace activity system is likely to dominate, even though relatively low failure rates and the “failure to fail” phenomenon have been reported (64). Although the prototypical intern concept may be less relevant to OSCE than WBA because of standardization of encounters and marksheets, acknowledging, and discussing prototypical intern qualities in standard setting may help drive fairer assessment. We believe that the contradictions have not been resolved, but rather clarified, potentially assisting further research. There may not be a “final” theory, but a clear message emerges: assessor judgements balance potentially conflicting perspectives that should be acknowledged and discussed in assessor training, standard setting, and calibration. Future research should investigate the identified contradictions.

There are implications for both marksheet design and assessor training. Marking rubrics may reflect what learners are “taught” but not necessarily the expectations of their imminent clinical service roles, suggesting a potential disconnect between achieving program learning outcomes and working in the clinical environment. The OSCE may currently present patient care as a set of individual tasks, whereas healthcare is being conceptualized increasingly as a team activity (65). Would marksheets that are more aligned with clinician constructs improve utility and compliance amongst predominantly clinical assessors? Should assessor training always include group discussion of how academic and clinical workplace constructs align as part of a more explicit “de-biasing” exercise? Should improving the fidelity of OSCEs to better reflect interprofessional healthcare teams not be possible, OSCEs may be better used as assessment “hurdles” that complement an increased emphasis on workplace-based assessment methods. These findings support calls for a review of the role of the OSCE as a clinical assessment tool (52).

## Limitations

Less experienced assessors were less represented because they were more difficult to recruit to interview sessions due to less flexible workload in their clinical service and specialty training requirements and a preference by medical schools for utilizing experienced assessors where available. Therefore, the perspectives of less experienced assessors may be under-represented. “Volunteer bias” is also possible, where assessors who follow medical school examiner training guidelines volunteered to be a part of this study—and “rogue examiners” did not volunteer. Hence, the statement that assessors usually followed institutional rules and guidelines for the OSCE may be overrepresented in this work. Additionally, the authors are insider researchers, and this could serve as potential bias, however, trustworthiness and credibility of the findings were enhanced through member checking and analytical group confirmations.

## Conclusion

Clinical assessment judgements in exit-level medical school OSCEs are complex, with individual assessors balancing perspectives from two different but interacting constructs that overlap and compete. The first is that of the academic system which is more task-oriented, emphasizing knowledge, skills and behaviors, based on achieving agreed graduate outcomes. The second is that of the clinical workplace, where graduates will soon have defined roles and responsibilities within a clinical team. The balance of the influence of these activity systems on judgements varies for individual assessors. Less experienced assessors tend to follow the academic rules listed in marksheets. More clinically engaged and more experienced assessors tend to fall back on a heuristic, mental construct of a “prototypical intern,” to calibrate judgements, particularly, in difficult situations. This heuristic is based on personal clinical experience and discussions with workplace peers, emphasizing professional attributes and trust, and may lead to a form of confirmation bias, that dominates thinking when candidates are “borderline;” time is pressured; or assessors are more experienced. Further research is needed to explore whether designing assessment marksheets and assessor training to more closely align the two systems decreases the cognitive load and increases the validity of assessor decision making. Designing marking frameworks should consider the possible introduction or amplification of unconscious biases. Further, assessor training may benefit from explicit “de-biasing” by aiming to increase awareness of a heuristic that is shared by assessors and caution against over-reliance on this strategy, thereby reducing unconscious bias.

## Data Availability Statement

The original contributions presented in the study are included in the article/Supplementary Material, further inquiries can be directed to the corresponding author.

## Ethics Statement

The studies involving human participants were reviewed and approved by the James Cook University Human Research Ethics Committee under Permit H6833. In addition, all participating schools obtained ethics approval from their local Ethics Committee. The participants provided their written informed consent to participate in this study.

## Author Contributions

BM-A, RH, and KD'S conceived the study and conducted the focus groups. BM-A, KJ, RH, KD'S, and SS advised on data analysis and interpretation. BM-A, RH, KD'S, SS, AC, and RT contributed to writing the original draft of the manuscript. All authors facilitated collection of data, reviewed, edited, and accepted the final version of the manuscript.

## Conflict of Interest

The authors declare that the research was conducted in the absence of any commercial or financial relationships that could be construed as a potential conflict of interest.

## Publisher's Note

All claims expressed in this article are solely those of the authors and do not necessarily represent those of their affiliated organizations, or those of the publisher, the editors and the reviewers. Any product that may be evaluated in this article, or claim that may be made by its manufacturer, is not guaranteed or endorsed by the publisher.
