# Supplementary material for: “Could You Work in My Team?”: Exploring How Professional Clinical Role Expectations Influence Decision-Making of Assessors During Exit-Level Medical School OSCEs
Source: Front Med (Lausanne). 2022 May 6;9:844899. doi: 10.3389/fmed.2022.844899 (PMC9120654; doi:10.3389/fmed.2022.844899)
Supplement: Supplementary file 1 [file Data_Sheet_1.docx]

## Supplementary Material 1 – Focus Group Questions

At the start of the FG, the aim of the research project was presented by the Moderator - Previous research work has raised the concept of a ‘prototypical (mythical) intern’.

- How does this concept fit with your approach when assessing final year medical students’ clinical performance?
- What image do you have of a competent medical graduate in Australia/New Zealand when you commence clinical assessment?
  - What is this based on?
- What would you look for in a candidate’s clinical work/performance to help decide how close he or she is to this level of performance?
  - What specific competencies do you look for? (additional probes if participants are stuck – interpersonal skills, specific procedural skills, professionalism, application of knowledge)
- How would you know if you could **trust** or **rely on** a candidate to be a working member of your clinical team?
  - What would make you feel concerned about their performance?
- What differences would you allow between performances of a final year medical student and a recent graduate? - (At the start of this question, the moderator will achieve a shared understanding with participants about who a minimally competent candidate is)
  - What is essential, what can you overlook?
  - How would this change for a minimally competent candidate?
- Based on all the discussions about the ‘prototypical (mythical) intern’, which words would you use to describe a candidate’s performance on a five-point clinical competence continuum scale?
  - For example, from unsafe to graduate to excellent, which words would you use to fill in the gaps?
  - How would this wording change when assessing a single task (e.g. history taking) in comparison to overall global performance?
