# Supplementary material for: “Could You Work in My Team?”: Exploring How Professional Clinical Role Expectations Influence Decision-Making of Assessors During Exit-Level Medical School OSCEs
Source: Front Med (Lausanne). 2022 May 6;9:844899. doi: 10.3389/fmed.2022.844899 (PMC9120654; doi:10.3389/fmed.2022.844899)
Supplement: Supplementary file 2 [file Data_Sheet_2.docx]

## Supplementary Material 2: Participants’ Profile

| **Participant Number** | **ID in Transcript** | **Sex** | **Roles/Experience** | **Years of Experience** |
| --- | --- | --- | --- | --- |
| P01 | Exp-M-P01 | M | Psychiatrist, Academic (assessor) | 15 |
| P02 | Exp-M-P02 | M | Obstetrician Gynaecologist, Academic (assessor) | 25 |
| P03 | Exp-F-P03 | F | General Practitioner/Senior Medical Educator | 18 |
| P04 | Exp-F-P04 | F | General Practitioner/Medical Educator | 10 |
| P05 | Exp-M-P05 | M | Medical Educator/Assessment/Anaesthetist | 45 |
| P06 | Exp-M-P06 | M | Clinician/Medical Educator | 15 |
| P07 | Exp-F-P07 | F | General Practitioner/Medical Educator | 10 |
| P08 | Exp-M-P08 | M | Rural Generalist Practitioner/Medical Educator/Assessor | 12 |
| P09 | Exp-F-P09 | F | Intensive Care Clinician | 7 |
| P10 | Exp-M-P10 | M | Endocrinologist/Academic/Assessor | 10 |
| P11 | Exp-F-P11 | F | Clinician/Assessor | 9 |
| P12 | Exp-M-P12 | M | Surgeon/Assessor | 20 |
| P13 | Exp-M-P13 | M | Surgeon/Assessor | 20 |
| P14 | Exp-M-P14 | M | Intensive Care Clinician/Assessor (clinical studies) | 12 |
| P15 | Less-F-P15 | F | Clinician/Associate Clinical Lecturer | 3 |
| P16 | Exp-M-P16 | M | Obstetrician Gynaecologist/College assessor | 12 |
| P17 | Less-M-P17 | M | Medical administrator/Medical Educator/Assessor | 1 |
| P18 | Less-F-P18 | F | Emergency Registrar | 2 |
| P19 | Less-M-P19 | M | Medical Registrar | 1 |
| P20 | Less-M-P20 | M | General Practitioner | 4 |
